# Complete Genome Sequence of a Psychrotolerant Denitrifying Bacterium, *Janthinobacterium svalbardensis* PAMC 27463

**DOI:** 10.1128/genomeA.01178-17

**Published:** 2017-11-16

**Authors:** Yong-Joon Cho, You-Jung Jung, Soon Gyu Hong, Ok-Sun Kim

**Affiliations:** Korea Polar Research Institute, Incheon, Republic of Korea

## Abstract

We report here the complete genome sequence of *Janthinobacterium svalbardensis* PAMC 27463 isolated from a freshwater lake on Barton Peninsula on King George Island, Antarctica. The genome consists of a chromosome with 6,274,078 bp which contains 5,585 genes, including 121 RNA genes.

## GENOME ANNOUNCEMENT

The species *Janthinobacterium svalbardensis*, a rod-shaped, Gram-negative, psychrotolerant bacterium, was previously reported from glacier ice samples in the Arctic (1). It is known to release a violacein-like pigment which defends against radiation and shows antimicrobial and anticancer activities (1–4). We isolated and sequenced *Janthinobacterium svalbardensis* PAMC 27463 from a freshwater lake on Barton Peninsula on King George Island, Antarctica (62°14′19.7″S, 58°44′41.7″W).

Genome sequencing was performed using PacBio RSII technology (Pacific Biosciences) using a 20-kb library, and the result yielded 983.7 Mb of sequence reads. *De novo* assembly was performed by SMRT Analysis version 2.3.0 (5), using HGAP2, with default parameters. The assembly generated only one contig with 136.84× coverage, and the circularization of this contig was executed with Circlator (6). Functional annotation was done using the NCBI Prokaryotic Genome Annotation Pipeline (PGAP). Clustered regularly interspaced short palindromic repeats (CRISPRs) were predicted using the CRISPR recognition tool (7), and prophage regions were identified by PHASTER (8).

The genome consists of one circular chromosome of 6,274,078 bp with 62.15% G+C content. A total of 5,585 genes were predicted in the genome of this strain, 5,360 of which are protein-coding genes, and the total length of coding regions is 5,495,121 bp. Of the protein-coding genes, 3,923 were assigned a putative function, and the remaining ones were annotated as hypothetical proteins. The genome also has 25 rRNAs and 92 tRNA genes. Two intact prophage regions were found, but no CRISPR was found in this genome.

Unexpectedly, the *vioABCDE* operon, which contains the genes responsible for the synthesis of the violacein pigment, was not found in the genome, but some species are not considered to have this gene set (9). The genome shows several enzymes involved in denitrification processes. The strain would reduce the nitrate from the extracellular environment by nitrate transporters (CNX70_12890, CNX70_14170, CNX70_14385, CNX70_16780, and CNX70_16810) to nitrite using nitrate reductase proteins (CNX70_14400, CNX70_14405, CNX70_14410, and CNX70_14415). The produced nitrite can be reduced to nitric oxide by the nitrite reductase proteins NirK (CNX70_19440, CNX70_20610, and CNX70_25620) and NirS (CNX70_15420 and CNX70_15425, CNX70_17105 and CNX70_17110, and CNX70_21255 and CNX70_21260). The genome also encodes nitric oxide reductase (CNX70_25610), but the nitrous oxide reductase was not found.

This strain may produce some of organic nitrogen compounds in an amino acid polymer, cyanophycin, using cyanophycin synthase (CNX70_22610 and CNX70_22615) and cyanophycinase (CNX70_25830). The stored cyanophycin can be used as a nitrogen source. Also, the genome encodes proteins for the biosynthesis of poly-beta-hydroxybutyrate (PHB), a kind of bioderived and biodegradable plastic. The strain may produce the PHB from acetyl-coenzyme A (acetyl-CoA) using acetyl-CoA C-acyltransferase (CNX70_16215), poly-beta-hydroxybutyrate polymerase (CNX70_14245), and polyhydroxyalkanoic acid system protein (CNX70_06405). Also, the genome harbors genes for PHB degradation (CNX70_01625, CNX70_01835, and CNX70_10875), and the strain may use the PHB as a carbon source.

This is the first report of a complete genome sequence of the *Janthinobacterium svalbardensis* species. In addition to its known properties, this bacterium is predicted to be able to produce and degrade the biodegradable PHB, and it can play a role in biotechnology applications.

### Accession number(s).

The genome sequence of *Janthinobacterium svalbardensis* PAMC 27463 was deposited in GenBank under the accession number CP023422.
